# Crystal structure of the plant receptor-like kinase TDR in complex with the TDIF peptide

**DOI:** 10.1038/ncomms12383

**Published:** 2016-08-08

**Authors:** Junko Morita, Kazuki Kato, Takanori Nakane, Yuki Kondo, Hiroo Fukuda, Hiroshi Nishimasu, Ryuichiro Ishitani, Osamu Nureki

**Affiliations:** 1 Department of Biological Sciences, Graduate School of Science, The University of Tokyo, 2-11-16 Yayoi, Bunkyo-ku, Tokyo 113-0032, Japan; 2 JST, PRESTO, 2-11-16 Yayoi, Bunkyo-ku, Tokyo 113-0032, Japan

## Abstract

In plants, leucine-rich repeat receptor-like kinases (LRR-RKs) perceive ligands, including peptides and small molecules, to regulate various physiological processes. TDIF, a member of the CLE peptide family, specifically interacts with the LRR-RK TDR to inhibit meristem differentiation into tracheary elements, and promotes cell proliferation. Here we report the crystal structure of the extracellular domain of TDR in complex with the TDIF peptide. The extracellular domain of TDR adopts a superhelical structure comprising 22 LRRs, and specifically recognizes TDIF by its inner concave surface. Together with our biochemical and sequence analyses, our structure reveals a conserved TDIF-recognition mechanism of TDR among plant species. Furthermore, a structural comparison of TDR with other plant LRR-RKs suggested the activation mechanism of TDR by TDIF. The structure of this CLE peptide receptor provides insights into the recognition mechanism of the CLE family peptides.

## Abstract (web summary)

The TDF peptide interacts with the leucine-rich repeat receptor-like kinase TDR to regulate meristem differentiation in plants. Here, the authors solve the structure of the extracellular domain of TDR in complex with TDIF and propose a mechanism for TDIF recognition.

## Graphical abstract

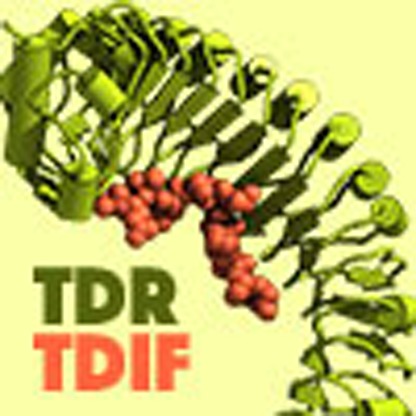

In plants, small secreted polypeptide hormones mediate the signalling pathways between cells, and regulate various physiological functions, including the immune response, differentiation and growth control^1^. The CLAVATA3 (CLV3)/endosperm surrounding region-related (CLE) family peptides, encoded by *CLE* genes, are the most thoroughly studied family among these small peptide hormones. The *CLE* family genes have been found in diverse plant species, including dicots, monocots, conifers, mosses and green algae^2^,^3^,^4^. They are expressed in various tissues by developmental and environmental cues and regulate numerous biological processes, including the proliferation and differentiation of stem cells, and the development of the meristem, the vascular system and the embryo^5^,^6^. In *Arabidopsis*, 32 *CLE* family genes have been identified, and all of them encode 12–13 amino-acid peptides with the conserved CLE domain at their C-termini^2^,^7^. The CLE peptides are secreted into the apoplast, after proteolytic processing and post-translational modifications. They mainly interact with the extracellular regions of membrane associated leucine-rich receptor kinases (LRR-RKs), thereby triggering intracellular signalling cascades^2^,^8^. Several ligand–receptor pairs of the CLE family peptides have been identified, including CLV3–CLV1 and CLE9/10–BAM1 (ref. 6).

Tracheary element differentiation factor (TDIF), a CLE family peptide, was first isolated from the xylogenic cell culture of *Zinnia elegans*. TDIF consists of 12 amino acids including two hydroxyproline residues, and is encoded by the *CLE41* and *CLE44* genes in *Arabidopsis*^9^. TDIF is secreted from the phloem and is perceived by its specific receptor, TDR/PXY (TDIF receptor/phloem intercalated with xylem), which is located on the procambial cell membrane^8^. PXY was originally identified as the component that maintains the cell polarity required for the orientation of cell division, during vascular development in the vascular meristem^10^. TDR belongs to the LRR-RK class XI subfamily, and consists of the extracellular LRR domain and the cytoplasmic kinase domain^10^. TDR recognizes TDIF by its extracellular LRR domain, and activates the intracellular GSK3 pathway to suppress the transcription factor BES1, which promotes cellular differentiation into tracheary elements^11^. Simultaneously, TDR activation enhances the transcription of the homeobox genes *WOX4* and *WOX14*, which promote procambial cell proliferation^12^,^13^. Furthermore, the tissue-specific co-overexpression of the TDR–TDIF pair increases wood formation by twofold in the aspen tree, suggesting that the modulation of the TDR–TDIF pathway can contribute to biomass engineering^14^.

Recently, several crystal structures of plant LRR-RKs in complex with their ligands were reported. The crystal structures of BRI1 and PSKR in complex with brassinolide and phytosulfokine, respectively, revealed the recognition mechanism of phytohormones by LRR-RKs (refs 15, 16, 17). Furthermore, the crystal structures of FLS and PEPR1 in complex with the bacterial flagella-derived peptide flg22 and the self-derived danger signal Pep1, respectively, demonstrated the epitope-recognition mechanism by LRR-RKs involved in the immune systems of plants^18^,^19^. However, the molecular mechanism of the CLE family peptide recognition by LRR-RKs has remained elusive, due to the lack of the crystal structures of LRR-RKs in complex with CLE peptides. Here, we report the crystal structure of the TDR–TDIF complex. In conjunction with the structure-guided functional analysis, our results provide structural insights into the mechanism of TDIF recognition by TDR.

## Results

### Structure determination

**Figure 1:**
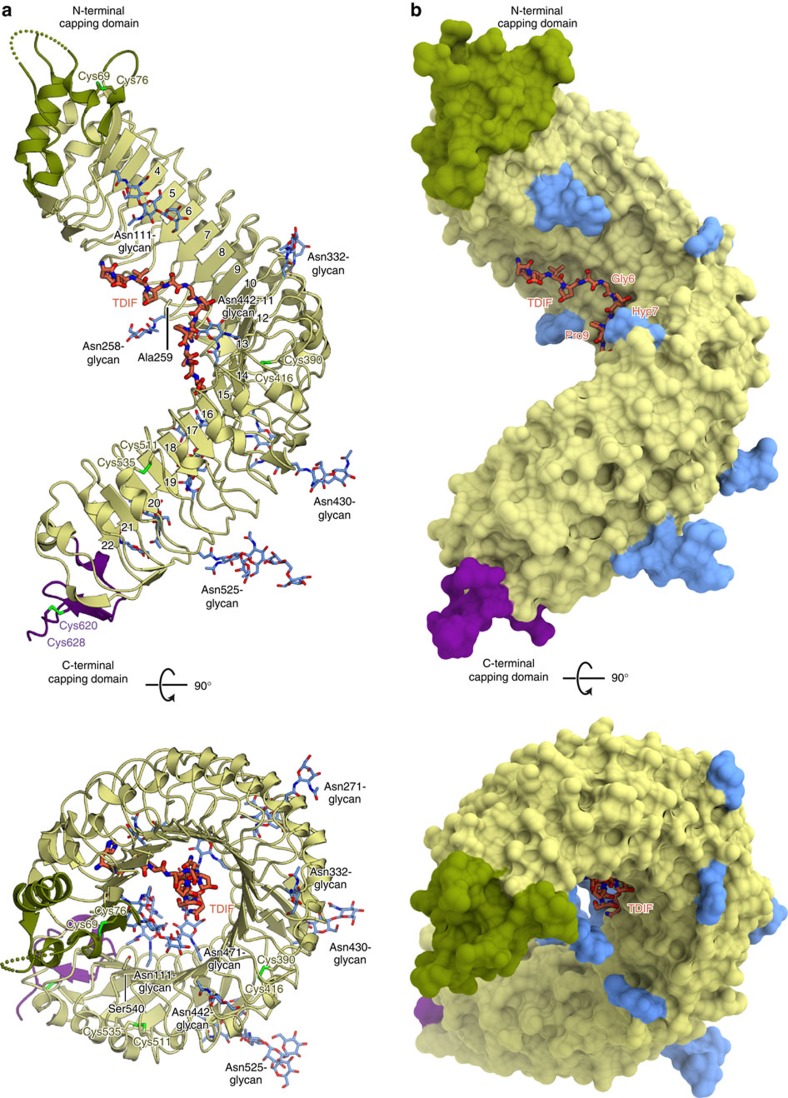
Overall structure of the TDR–TDIF complex. (**a**,**b**) Ribbon representation (**a**) and surface representation (**b**) of the crystal structure of the extracellular LRR domain of TDR in complex with TDIF. TDIF, *N*-linked glycans and Cys residues that form disulfide bonds are shown as orange, blue and yellow-green sticks, respectively. Two residues mutated from cysteines (Ala259 and Ser540) are also shown as sticks. The N-terminal capping domain is coloured dark green. The positions of the LRRs are indicated.

Initially, we prepared the extracellular domain (residues 31–631) of TDR from *A. thaliana*, using the Sf9-baculovirus expression system. We attempted the crystallization screening of the complex with a synthesized TDIF peptide (HEV(Hyp)SG(Hyp)NPISN), but no crystals were obtained. To improve the solution behaviour of the TDR extracellular domain, we introduced mutations to two less-conserved cysteine residues (C259A/C540S), and performed the crystallization screening using the lysine methylated C259A/C540S TDR mutant^20^. A pull-down assay using the biotinylated TDIF peptide confirmed that the lysine methylated C259A/C540S mutant has TDIF-binding activity comparable to that of the wild-type protein (Supplementary Fig. 1a). Furthermore, to measure the signalling activity of the C259A/C540S mutant, we reconstructed the TDIF–TDR–BIN2 pathway in *Nicotiana benthamiana* leaves with TDR or its mutants fused with the cyan fluorescent protein (TDR–CFP), together with BIN2 fused with the yellow fluorescent protein (BIN2–YFP), as previously described^11^ (Supplementary Fig. 1b,c). We confirmed that the C259A/C540S mutant TDR localizes to the plasma membrane, similarly to wild-type TDR (Supplementary Fig. 1d). Both the wild-type TDR and C259A/C540S mutant showed reduced fluorescence resonance energy transfer (FRET) efficiency of TDR–CFP with BIN2–YFP, on the TDIF peptide application, indicating that C259A/C540S mutant TDR possesses TDIF responsiveness comparable to that of the wild-type protein (Supplementary Fig. 1e). In addition, the biotinylated TDIF peptide used for pull-down assays also reduced the FRET efficiency, indicating that the biotinylated TDIF peptide is biologically active (Supplementary Fig. 1f). Finally, we obtained the crystals of the TDR–TDIF complex, which diffracted X-rays up to 3.0 Å resolution. We determined the crystal structure at 3.0 Å resolution by molecular replacement, using the structure of the extracellular LRR domain of FLS2 (PDB ID: 4MN8) as the search model (Table 1, Fig. 1).

### Overall structure

The extracellular domain of TDR forms a twisted right-handed superhelix composed of 22 LRRs and N-terminal (residues 34–81) and C-terminal (residues 609–637) capping domains (Fig. 1 a,b). The N-terminal capping domain is assembled from α helices, a β strand and a disordered loop (residues 61–64), and is stabilized by a disulfide bond (Cys69–Cys76; Fig. 1a,b). The C-terminal capping domain is assembled from a 3_10_ helix and a β strand, and is stabilized by a disulfide bond (Cys620–Cys629; Fig. 1a). Plant LRR proteins typically contain repeat sequences of 20–29 amino acids with an LxxLxLxxNxGxIP consensus motif, where X represents any amino acid, and Leu is sometimes substituted by other hydrophobic residues, such as Phe or Val (ref. 21). The known plant LRR-RK structures share the inner concave surface, consisting of parallel β strands formed by the LxxLxL motif, while the outer convex surface consists of various secondary structures, including α helices, 3_10_ helices and short additional β strands^15^,^16^,^17^,^18^,^19^,^22^. The present crystal structure of TDR revealed that the repetition of 23–24 amino acids forms the 22 LRR domains (LRR1–LRR22), which compose the inner concave surface with the parallel β strands formed by the LxxLxL motif, and the outer convex surface with helices or loops formed by variable sequences following the GxIP motif (Supplementary Fig. 2). Two loops on the convex surface are stabilized through disulfide bonds formed between adjacent repeats (LRR13–LRR14 and LRR18–LRR19). In the crystal structure, we observed the electron densities for the 11 glycan chains attached to the Asn side chains in the LRR domains (Fig. 1a,b and Supplementary Fig. 2), although their biological functions are unknown.

### TDIF kink recognition by the TDR LRR domains

**Figure 2:**
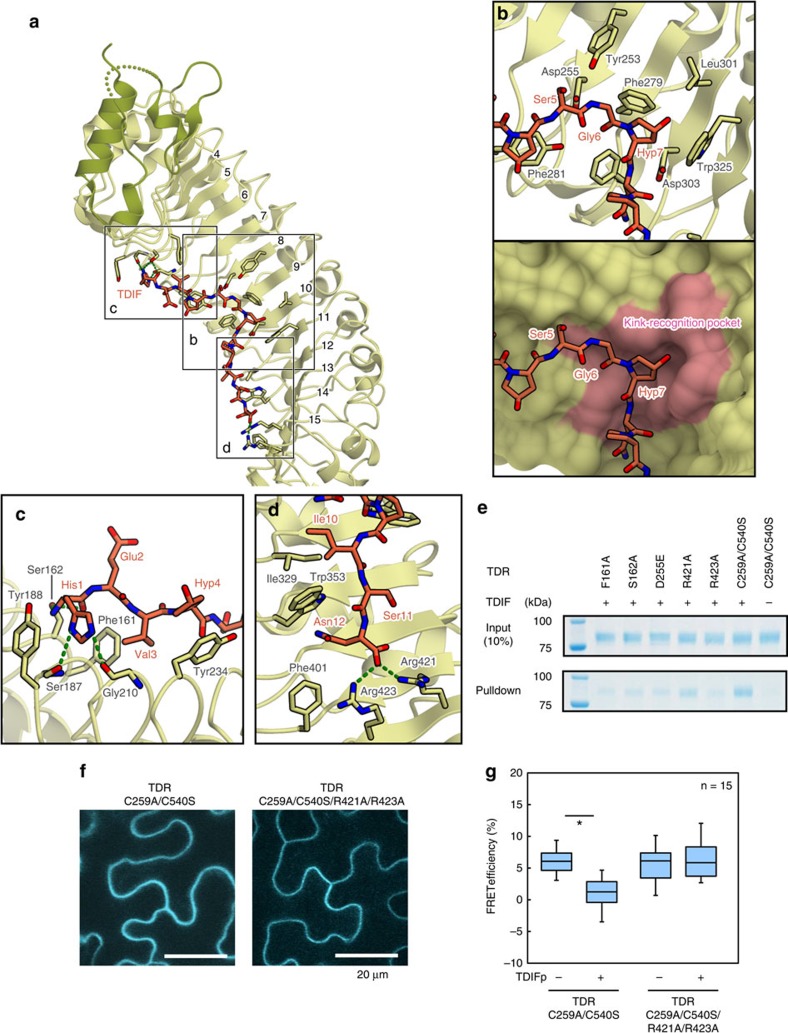
TDIF recognition by TDR. (**a**) Interaction between TDIF and TDR. The TDIF-interacting residues of TDR are shown in stick representations. TDIF and TDR are coloured orange and green, respectively. Hydrogen bonds are shown as dashed green lines. (**b**–**d**) Recognition of the middle part (residues 5–7) (**b**), the N-terminal part (residues 1–4) (**c**), and the C-terminal part (residues 10–12) (**d**) of TDIF by TDR. In **b**, TDR is shown in a ribbon representation (upper) and a surface representation (lower). The kink-recognition pocket is coloured pink. (**e**) Pull-down experiment using the biotinylated TDIF and TDR mutants. The C259A/C540S TDR mutant was used for crystallization, and other mutations were introduced to this construct. These TDR mutants were expressed and purified from Sf9 insect cells, and mixed with Streptavidin beads in the presence or absence of biotinylated TDIF. The bound proteins were eluted with SDS sample buffer and analysed by SDS–PAGE. See also Supplementary Fig. 1a. (**f**) Localization of TDR–CFP variants in *Nicotiana benthamiana* epidermis. Scale bar indicates 20 μm. (**g**) TDIF responses in TDR mutated variants. TDIF responses were evaluated by measuring FRET efficiencies between TDR–CFP and BIN2–YFP. The boxplot diagram displays FRET efficiencies in incubations with or without 5 μM TDIF for 30 min. Significant differences according to the Student's *t*-test are indicated by asterisks (*P*<0.01; *n*=15).

The electron density of TDIF is clearly defined on the concave surface of LRR4–LRR15 of TDR (Fig. 1, Supplementary Fig. 3). TDIF adopts an extended conformation, with a kink around Gly6–Hyp7 at the middle of the peptide. The backbone of the TDIF peptide forms van der Waals interactions with the bulky side chains of TDR, such as Phe and Trp (Fig. 2a–d). The interactions between TDIF and TDR can be divided into three parts: the N-terminal part (His1–Hyp4), the middle part (Ser5–Hyp7) and the C-terminal part (Asn8–Asn12; Fig. 2a–d).

In the middle part, the kink around Gly6–Hyp7 is recognized by the pocket formed by Asp255 (LRR8), Phe279 (LRR9), Phe281 (LRR9) and Trp325 (LRR11) of TDR, in a shape-complementary manner (Fig. 2b). To verify the importance of the TDIF kink recognition by TDR, we performed pull-down assays using biotinylated TDIF peptides. The results showed that the D255E mutation of TDR, which may change the size of the kink-recognition pocket, reduced its TDIF binding, as compared with those of wild-type TDR and the C259A/C540S mutant (Fig. 2e, Supplementary Fig. 1a). Furthermore, a previous study showed that the G6A mutation abolished the TDIF activity^9^. These results suggest that the kink formation in TDIF and the kink-recognition pocket of TDR are critical for the TDR–TDIF interaction. A previous study reported that the post-transcriptional hydroxylation of Hyp7 is dispensable for the TDIF activity^9^. Consistently, the hydroxyl group of Hyp7 is not recognized by TDR, although the other moieties of Hyp7 form van der Waals interactions with the side chains of Phe279, Leu301 and Trp325 of TDR. Taken together, the central kink of the TDIF peptide is recognized by the kink-recognition pocket formed by the TDR LRR domains, through shape complementarity.

### Sequence-specific recognition of TDIF by TDR

In the N-terminal part, the imidazole nitrogen of His1 of TDIF hydrogen bonds with the main-chain carbonyl groups of Ser187 (LRR5) and Gly210 (LRR6), and the N-terminal amino group and the carbonyl group of His1 of TDIF form further hydrogen bonds with the hydroxyl group of Ser162 (LRR4) of TDR. The Val3 side chain of TDIF interacts with the hydrophobic surface around Phe161 (LRR4) of TDR (Fig. 2c). The results of the pull-down assay demonstrated that the F161A and S162A mutations of TDR decreased the TDIF binding, suggesting the importance of these interactions with the N-terminal part (Fig. 2e, Supplementary Fig. 1a). Moreover, a previous study reported that the H1A and V3A mutations in TDIF diminished the TDIF activity *in planta*^9^, suggesting the biological importance of the interactions observed in the crystal structure. In contrast, the side chains of Glu2 and Hyp4 are not recognized by TDR; the Glu2 side chain is disordered, while Hyp4 is solvent-exposed in the present crystal structure. These observations are consistent with the previous study showing that both the E2A mutation and the Hyp4 to Pro substitution in TDIF do not affect the inhibitory activity against the tracheary element differentiation^9^.

At the C-terminal part, the Asn12 side-chain carbonyl group of TDIF hydrogen bonds with the side chains of Arg421 and Arg423 of TDR (Fig. 2d). The pull-down assay revealed that the R423A mutation reduced the TDIF-binding activity, while the R421A mutation slightly reduced it (Fig. 2e). Consistent with the results from the *in vitro* assay, the R421A/R423A mutation abolished the responsiveness to TDIF in the reconstructed TDIF–TDR–BIN2 pathway in *Nicotiana benthamiana* leaves, confirming the importance of these TDR–TDIF interactions in the cellular context (Fig. 2f,g). Moreover, a previous study showed that the N12A mutation of TDIF diminishes the TDIF activity^9^. These results suggest that the recognition of the C-terminal Asn residue of TDIF by Arg423 of TDR is critical for the TDR–TDIF signalling pathway *in planta*. The Phe161, Ser162, Arg421 and Arg423 residues are conserved among the TDRs from other plants, implying that the TDIF-recognition mechanisms are also conserved among plant species (Supplementary Fig. 4). Taken together, the N and C termini of TDIF are both recognized by TDR through sequence-specific hydrogen bonding interactions.

## Discussion

In this study, we determined the crystal structure of the extracellular domain of TDR in complex with its ligand, TDIF. In conjunction with the functional analyses, our results revealed the recognition mechanism of TDIF by TDR. While our manuscript was under revision, Zhang *et al*.^23^ reported the crystal structure of TDR–TDIF, which is essentially identical to our structure (root-mean-square deviation of 1.23 Å for 585 aligned Cα atoms; Supplementary Fig. 5), confirming the functional relevance of the TDIF-recognition mechanism of TDR. In addition, their genetic complementation assays in *Arabidopsis* affirmed that the C-terminal recognition of TDIF by the positive charges of Arg421 and Arg423 in TDR is required for the *in vivo* function of TDR, in agreement with the results of our *in vitro* and *in vivo* assays. Moreover, Zhang *et al*. showed that TDR forms a TDIF-dependent heterodimer with the extracellular LRR domain of SERK1, a homologue of BAK1 (also known as SERK3), thereby indicating that SERK1 acts as a co-receptor of TDR.

**Figure 3:**
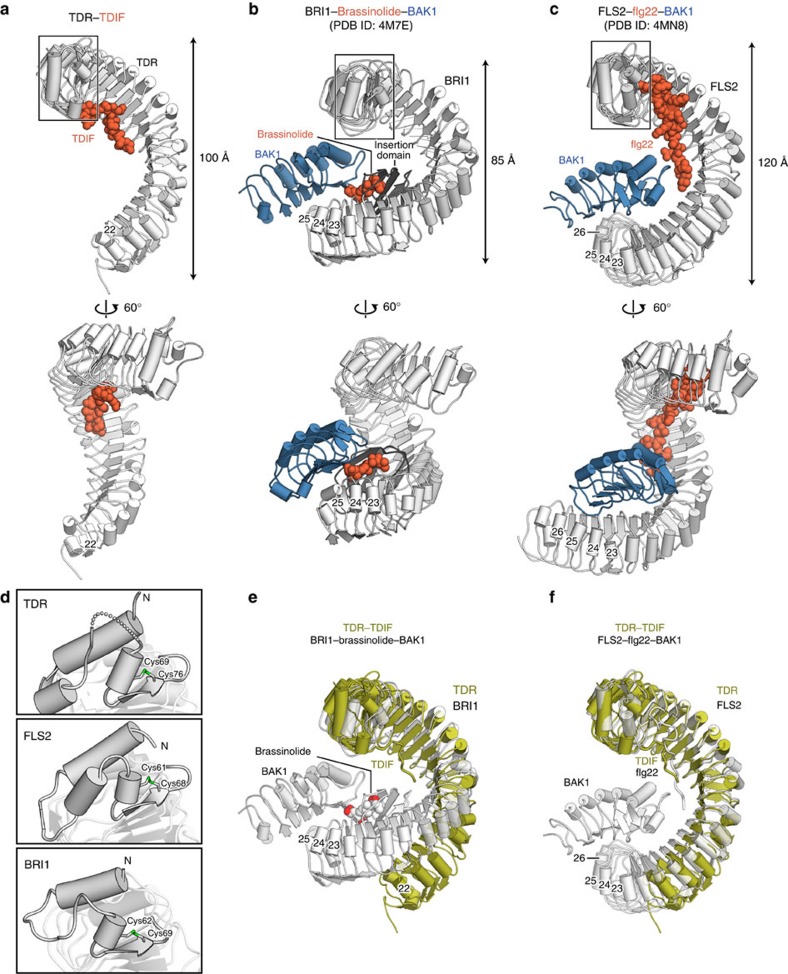
Structural comparison of the extracellular domains of plant LRR-RKs. (**a**–**c**) Crystal structures of the TDR–TDIF complex (**a**), the BRI1–brassinolide–BAK1 complex (**b**) and the FLS2–flg22–BAK1 complex (**c**). LRR-RKs, co-receptors and ligands are coloured white, blue and orange, respectively. The insertion domain of BRI1 is coloured dark grey. (**d**) N-terminal capping domains of TDR, FLS2 and BRI1. The disordered region (residues 61–64) is shown as a dashed line. Cys residues that form disulfide bonds are shown as sticks. (**e**,**f**) Superimposition of TDR onto the BRI1–brassinolide–BAK1 complex (**e**) and the FLS2–flg22–BAK1 complex (**f**). TDR and the LRR-RK–ligand–BAK1 complexes are shown in olive and white, respectively.

A comparison of the TDR–TDIF complex structure with those of known plant LRR-RKs and their ligand complexes revealed the conserved structural features among the plant LRR-RK family members. The twisted superhelical structures and the N-terminal capping domain of the LRR domain of TDR are commonly observed in the other plant LRR-RK structures, including BRI1 and FLS2 (refs 15, 16, 18; Fig. 3a–d). In the LRR domains of these structures, the LxxLxL motif specific to the plant LRR-RKs is composed of parallel β sheets, with their Leu residues facing towards the inner side of the molecule, to form the hydrophobic core in the proteins. Furthermore, the N- and C-terminal capping domains contribute to structural stabilization, by preventing the exposure of the hydrophobic core to the solvent^21^. Nonetheless, there are also notable structural differences between TDR and the other LRR-RKs. The LRR-RKs FLS2 and BRI1 form ligand-dependent heterodimers with the co-receptor BAK1, mediated by their ligands, flg22 and brassinolide, respectively. In these structures, the ligands are sandwiched between the receptor and the co-receptor, thereby serving as molecular glue^18^,^24^ (Fig. 3b,c). In FLS2, LRR23–LRR26 are involved in the interaction with BAK1, while in BRI1, both LRR23–LRR25 and the insertion domain between LRR21 and LRR22 are involved in the ligand-mediated heterodimerization with BAK1. Notably, these structural features are absent in the LRR domains of TDR; the LRR domains of TDR are shorter than those of FLS2 and BRI1, and lack the insertion domain (Fig. 3e,f). Moreover, the LRR domains of TDR and BRI1 have different curvatures. These structural differences suggest that TDR–TDIF form a heterodimer with SERK1, which shares 72% sequence identity with BAK1 in *Arabidopsis thaliana*, in a manner distinct from those in the other BAK1-mediated receptors (that is, BRI1–brassinolide–BAK1 and FLS2–flg22–BAK1; Fig. 3). Further structural studies will be required to elucidate the mechanism by which TDR forms a heterodimer with SERK1 in a TDIF-dependent manner.

The present crystal structure provides structural insights into the common recognition mechanism of the CLE family peptide hormones by their receptors. The kink-forming residues of TDIF (Gly6 and Hyp7) are highly conserved among the CLE family peptides (Supplementary Fig. 6a). Previous studies reported that the G6A mutations in other CLE peptides also abrogate their activities, as observed in the case of TDIF (refs 9, 25). Moreover, the residues in the kink-recognition pocket of TDR (Asp255, Phe279 and Trp325) are well conserved among the CLE family peptide receptors, such as CLV1, BAM1/2/3 and SKM1 (Supplementary Fig. 6b). These observations suggest that the accommodation of the kinked peptide by the receptor pocket is the general interaction mechanism for the CLE family ligands and their receptors. In contrast, a previous study reported that several CLE family peptides other than TDIF do not interact with TDR (ref. 8). The present crystal structure explains the mechanism by which TDR discriminates TDIF from other CLE peptides. At the C-terminal part of the molecular interface between TDR and TDIF, the Arg421 side chain of TDR hydrogen bonds with the Asn12 side chain of TDIF. In the CLV3 peptide, this Asn12 residue is replaced with His, while Arg421 of TDR corresponds to Lys413 of CLV1, an LRR-RK receptor for the CLV3 peptide. Thus, it is possible that the interaction at the C-terminal part is important for the discrimination of the orthogonal ligand–receptor pairs. Consistently, Arg421 is also conserved in other LRR-RKs, such as BAM1/2/3 and SKM1, which recognize the CLE peptides that possess Asn at position 12 (refs 26, 27, 28; Supplementary Fig. 5b). Furthermore, at the N-terminal part of the interface, the His1 side chain of TDIF is recognized by the main-chain carbonyl groups of Ser187 and Gly210 of TDR, and the Val3 side chain of TDIF interacts with the surface around Phe161 of TDR. These interactions can also explain the substrate specificity of TDR. A previous study reported that CLV3, CLE9, CLE19 and CLE46 do not interact with TDR (ref. 8). His1 of TDIF is replaced with Arg in CLV3, CLE9 and CLE19, indicating that the longer side chain of Arg cannot hydrogen bond with the main-chain carbonyl groups of TDR in a similar manner. In CLE46, Val3 of TDIF is replaced with His, showing that the bulky side chain of His cannot interact with the TDR surface around Phe161. In contrast, a previous study reported that CLE42 can act as a ligand of TDR in *Arabidopsis*^29^. Glu2 of TDIF is replaced with Gly in CLE42, consistent with the fact that the Glu2 side chain is not recognized by TDR. Collectively, the present crystal structure suggests that the central kink of the CLE peptides, including TDIF, offers a common platform for the recognition by the CLE peptide receptors, while the N- and C-terminal parts provide the sequence-specific readout for the cognate CLE receptors.

In conclusion, our structural and functional data revealed the recognition mechanism of TDIF by TDR, and provided insights into the recognition mechanism of the CLE family peptides by their cognate receptors. These findings may help pave the way for the rational engineering of the TDR–TDIF axis, to improve biomass production.

## Methods

### Protein expression and purification

DNA fragments encoding the extracellular LRR domain of TDR from *Arabidopsis thaliana* (residues 31–631) were amplified by PCR using pDONR-221-TDR as templates, PrimeSTAR MAX DNA polymerase (Takara Bio Inc) and the following primers: 5′- ATTGTGGGTTCAGCGAAGTTTTCACCTCAACTCTTGTCTCTC -3′ and 5′- AACTTCCAGGCCGCTATCAGAATTGCAAGGTTTTCCGAC -3′. The PCR products were inserted into pFastBac1 vector (Invitrogen), which had been modified to contain a N-terminal Hemolin peptide from *Hyalophora cecropia* (residues 1–18) and a C-terminal HRV3C protease cleavage site followed by a 10 × His tag (pHem-HRV3C- His10-TDR). To improve the solution behaviour of TDR, two less-conserved cysteine residues were mutated (C259A/C540S) by a PCR-based method using pHem-HRV3C-His10-TDR as templates, PrimeSTAR MAX DNA polymerase (Takara Bio Inc) and the following primers: C259A, 5′- CTTTGACGTTTCCAATGCCAGCCTCTCTGGTTC -3′ and 5′- GAACCAGAGAGGCTGGCATTGGAAACGTCAAAG -3′; C540S, 5′- CTTCTCTCTTTGAATCTCAGCCAAAATCATC -3′ and 5′- CTTCTCGCAATGTCCGATG -3′. The DNA sequences were verified by DNA sequencing. Baculoviruses were generated according to the manufacturer's instructions for expression in Sf9 insect cells, and baculovirus-infected Sf9 cells were cultured in Sf900II medium (Invitrogen) at 27 °C for 72 h and then harvested. The culture supernatant was incubated with Ni Sepharose excel (GE Healthcare) resin at 4 °C overnight, and the resin was then washed with buffer consisting of 20 mM Tris-HCl, pH 7.5, 500 mM NaCl, and 20 mM imidazole on an Econo-Column (Bio-Rad). The bound protein was eluted with buffer consisting of 20 mM Tris-HCl, pH 7.5, 500 mM NaCl and 500 mM imidazole, and then dialyzed against 20 mM Tris-HCl, pH 7.5 and 150 mM NaCl to remove the imidazole. The protein was incubated with Talon cobalt affinity resin (Clontech). The resin was washed with buffer supplemented with 20 mM imidazole, and then the protein was eluted with buffer supplemented with 300 mM imidazole. The eluted protein was dialyzed against 20 mM Tris-HCl, pH 7.5, 150 mM NaCl and 20 mM imidazole with HRV3C protease to cleave the C-terminal His tag, and then was passed through the Talon column again. The purified protein was digested with Endo H (NEB) at 20 °C overnight. To facilitate crystallization, the lysine residues of the deglycosylated TDR protein were methylated, as previously described^20^. In brief, the protein was incubated with the dimethylamine-borane (ABC) complex and formaldehyde in 50 mM HEPES, pH 7.5, at 4 °C overnight. The methylated TDR protein was further purified by gel filtration chromatography on a Superdex 200 Increase column (GE Healthcare), and concentrated to 3 mg ml^−1^ using an Amicon Ultra-4 filter (30 kDa molecular-weight cutoff; Millipore). The purity of the protein was assessed by SDS–polyacrylamide gel electrophoresis (SDS–PAGE) under nonreducing conditions, and the gels were stained with Simply Blue SafeStain (Invitrogen).

### Crystallization

A mixture of TDR and a chemically synthesized TDIF peptide (HEV(Hyp)SG(Hyp)NPISN, Eurofins Genomics), at a molar ratio of 1:1.5, was crystallized at 20 °C by the vapour diffusion method. Crystals were obtained by mixing 1 μl of protein solution (3 mg ml^−1^ TDR, 75 μM TDIF, 20 mM Tris-HCl, pH 7.5 and 150 mM NaCl) and 1 μl of reservoir solution (4 M sodium nitrate, 0.1 M sodium acetate trihydrate, pH 4.8 and 200 mM ammonium sulfate). Crystals were cryoprotected in reservoir solution supplemented with 6 M sodium nitrate, and were flash-cooled in liquid nitrogen.

### Structure determination

X-ray diffraction data were collected at 100 K on the beamline PXI at the Swiss Light Source, and processed using DIALS (ref. 30). The structure was determined by molecular replacement with MOLREP (ref. 31) using truncated FLS2 (residues 56–611, PDB ID 4MNA), in which the residues that did not align with TDR were removed, as the initial search model. Automated model building was performed with *Buccaneer*^32^, and the resulting model was manually completed with COOT (ref. 33). The refinement was performed using REFMAC (ref. 34) and PHENIX (ref. 35).

### Mutant protein preparation

The TDR mutants were prepared by a PCR-based method, using the expression vector encoding the TDR C259A/C540S mutant as the template PrimeSTAR MAX DNA polymerase (Takara Bio Inc) and the following primers: F161A, 5′- CTTAAAAGTCTTCAATGCGGCCAGCAACAACTTCG -3′ and 5′- CGAAGTTGTTGCTGGCCGCATTGAAGACTTTTAAG -3′; S162A, 5′- GTCTTCAATGCGTTCGCCAACAACTTCGAAGG -3′ and 5′- CCTTCGAAGTTGTTGGCGAACGCATTGAAGAC -3′; D255E, 5′- CAAATCTCAAGTACTTTGAAGTTTCCAATGCCAGC -3′ and 5′- GCTGGCATTGGAAACTTCAAAGTACTTGAGATTTG -3′; R421A, 5′- GCGAATCTCTATGGGCGTTTCGGAGTCAAAAC -3′ and 5′- GTTTTGACTCCGAAACGCCCATAGAGATTCGC -3′; R423A, 5′- CTCTATGGCGGTTTGCGAGTCAAAACAATCG -3′ and 5′- CGATTGTTTTGACTCGCAAACCGCCATAGAG -3′; R421A/R423A, 5′- CTCTATGGGCGTTTGCGAGTCAAAACAATCG -3′ and 5′- CGATTGTTTTGACTCGCAAACGCCCATAGAG -3′, and the sequences were verified by DNA sequencing. Baculovirus generation and protein expression were performed in the same manner as that for the TDR C259A/C540S mutant. The mutant proteins were purified using Ni Sepharose excel resin, in a similar manner to that for the TDR C259A/C540S mutant.

### Pull-down assay

The biotinylated TDIF peptide (2 μg) (HEV(Hyp)SG(Hyp)NPISN-GSGS-(Ahx)-Biotin, Eurofins Genomics) was mixed with 10 μl of Dynabeads M-280 Streptavidin (Life Technologies) beads at room temperature for 30 min. The beads were washed with 200 μl of wash buffer (20 mM Tris-HCl, pH 7.5, 150 mM NaCl) three times. To test the interaction between TDR and TDIF, the purified TDR protein was mixed with the biotinylated TDIF-bound beads in the presence of 0.1 mg ml^−1^ BSA, and then incubated at 4 °C for 2 h. The beads were then washed three times with 200 μl wash buffer. The bound proteins were eluted by boiling in SDS sample buffer and analysed by SDS–PAGE. Full images of SDS–PAGE gels are indicated in Supplementary Fig. 7.

### FRET analysis

Mutated TDR variants were produced by site-directed mutagenesis with the pDONR221 vector harbouring TDR. The coding sequences of BIN2 and the mutated TDR were transferred from the entry clones into the destination vectors by the LR recombination reaction, to generate 35 S-BIN2–YFP and pER8-TDR(mut)-CFP. These expression vectors were transformed into the *Rhizobium radiobacter* strain GV3101 MP90. The cultured agrobacterium suspended in infiltration buffer (10 mM MES, 10 mM MgCl_2_ and 150 μM acetosyringone, pH 5.7) was injected into *Nicotiana benthamiana* leaves with a 1 ml syringe (Terumo). After 2 days of infiltration, the tobacco leaf disks were treated with 10 μM estradiol for 18 h. After confirming YFP and CFP expression in the leaf epidermis, the FRET analysis was performed according to the acceptor photobleaching method, using an LSM-510 META confocal microscope (Carl Zeiss). First, we measured the CFP fluorescent signal intensities at the plasma membrane. Subsequently, BIN2–YFP was photobleached with 514 nm laser irradiation at the plasma membrane, and then we re-measured the CFP signal intensities. For the detection of the CFP fluorescent signal, a 458 nm excitation laser and a 477–520 nm emission filter were used. FRET efficiencies were calculated by comparing CFP fluorescent intensities before and after photobleaching.

### Data availability

Structures described in this manuscript have been deposited in Protein Data Bank under accession code 5GIJ. The authors declare that all other data supporting the findings of this study are included in the manuscript and its Supplementary Files or are available from the corresponding author upon request

## Additional information

**How to cite this article:** Morita, J. *et al*. Crystal structure of the plant receptor-like kinase TDR in complex with the TDIF peptide. *Nat. Commun.* 7:12383 doi: 10.1038/ncomms12383 (2016).

## Supplementary Material

Supplementary Information Supplementary Figures 1 - 7

## Figures and Tables

**Table 1:** Data collection and refinement statistics.

*Data collection*	
Beamline	SLS PX I
Wavelength (Å)	1.00
Space group	*P*3_1_21
Cell dimensions	
*a*, *b*, *c* (Å)	132.9, 132.9, 229.8
*α*, *β*, *γ* (°)	90.0, 90.0, 120.0
Resolution (Å)	103–3.00 (3.11–3.00)
*R*_pim_	0.053 (0.436)
*I*/σ*I*	8.8 (1.4)
Completeness (%)	99.8 (99.9)
Redundancy	9.7 (10.1)
CC(1/2)	0.995 (0.800)
	
*Refinement*	
Resolutions (Å)	57.6–3.00 (3.06–3.00)
No. of reflections	47,591
*R*_work_/*R*_free_	0.220/0.238
No. of atoms	
Protein	5,089
Solvent	0
*B*-factors (Å^2^)	
Protein	80.9
r.m.s. deviations	
Bond length (Å)	0.004
Bond angles (°)	1.210
Ramachandran plot	
Favoured region	94.42%
Allowed region	5.58%
Outlier region	0.00%

Values in parentheses are for the highest-resolution shell.
