# Supplementary material for: Crystal structure of the plant receptor-like kinase TDR in complex with the TDIF peptide
Source: Nat Commun. 2016 Aug 8;7:12383. doi: 10.1038/ncomms12383 (PMC4979064; doi:10.1038/ncomms12383)
Supplement: Supplementary Information — Supplementary Figures 1 - 7 [file ncomms12383-s1.pdf]

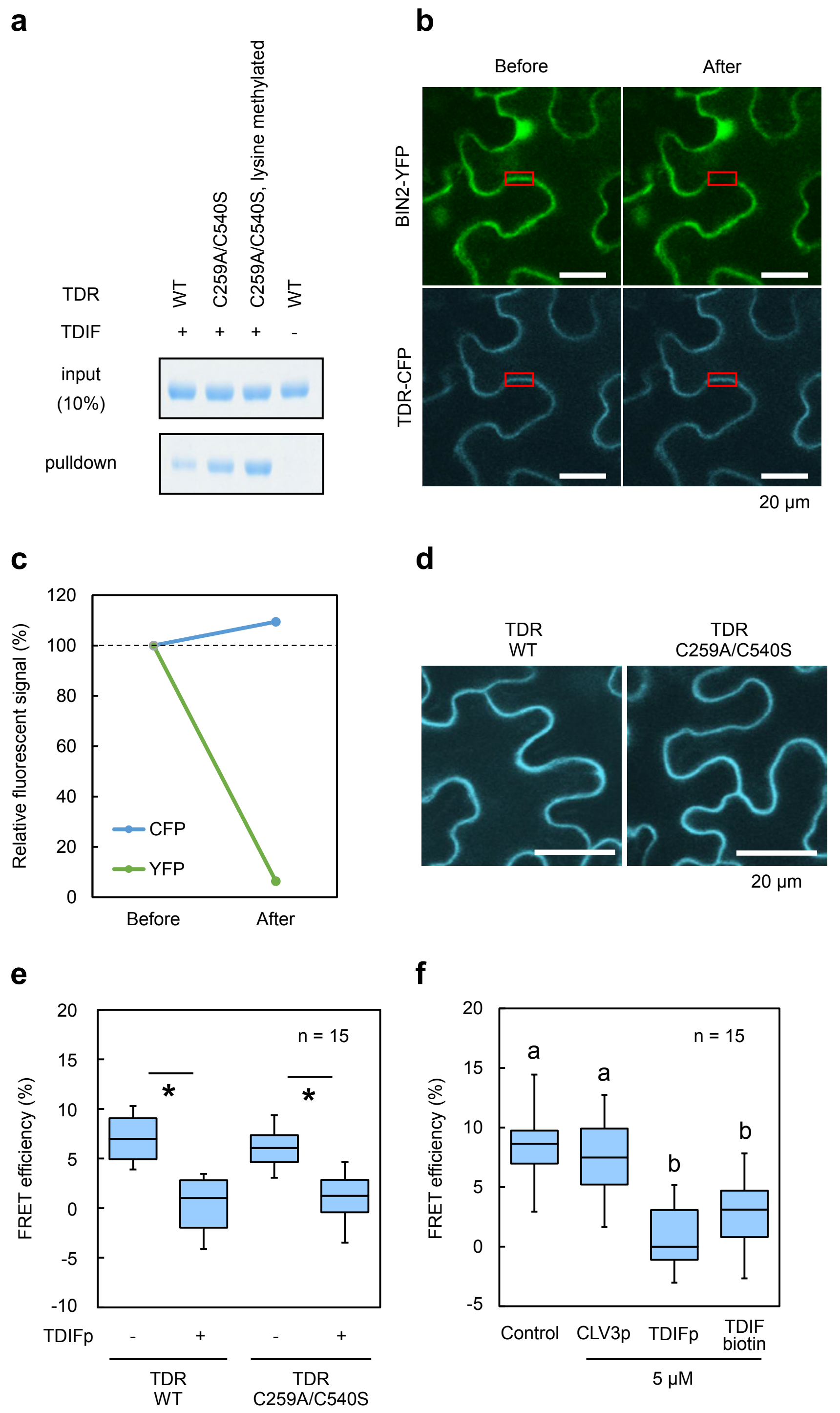

Supplementary Figure 1. Activities of TDR construct used for crystallization.

(a) Pull-down experiments between biotinylated-TDIF and TDR WT and the lysine methylated C259A/C540S TDR mutant. The wild type and C259A/C540S mutant of TDR were expressed and purified from Sf9 insect cells, and mixed with Streptavidin beads in the presence or absence of biotinylated TDIF. Bound proteins were eluted with SDS sample buffer and analyzed by SDS-PAGE. (b) Subcellular localization of BIN2–YFP and TDR–CFP in N. benthamiana, before and after photobleaching. Areas surrounded by red rectangles indicate the regions photobleached by the 514 nm laser. Scale bars indicate 20 μm. (c) Quantification of the fluorescent signals of BIN2–YFP and TDR–CFP in the red rectangle areas in (b). The relative fluorescent signals were calculated by comparison with the signal before photobleaching and are shown as a percentage (%). (d) Localization of TDR–CFP variants in Nicotiana benthamiana epidermis. Scale bars indicate 20 μm. (e) TDIF responses in the TDR C259A/C540S mutant. TDIF responses were evaluated by measuring FRET efficiencies between TDR–CFP and BIN2–YFP. The boxplot diagram displays the FRET efficiencies upon incubations with or without 5 μM TDIF for 30 min. Significant differences according to the Student's t-test are indicated by asterisks ($P < 0.01$; n = 15). (f) Bioactivities of synthetic peptides. TDIF activities of synthetic peptides were evaluated by measuring FRET efficiencies between TDR–CFP and BIN2–YFP. The boxplot diagram displays the FRET efficiencies with 5 μM synthetic peptides applied for 30 min. Significant differences ($P < 0.05$) are indicated by distinct letters (Tukey's test; n = 15).

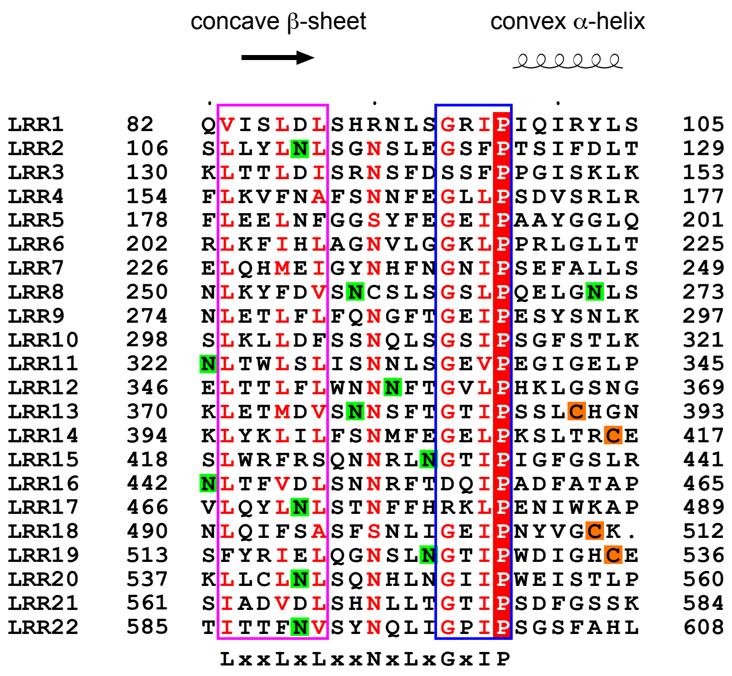

Supplementary Figure 2. Sequence alignment of LRRs of TDR.

The Cys residues that form disulfide bonds are indicated with an orange background, and the glycosylated Asn residues are indicated with a green background. The residues that form the concave β sheets and the convex α helices are enclosed in magenta and blue boxes, respectively.

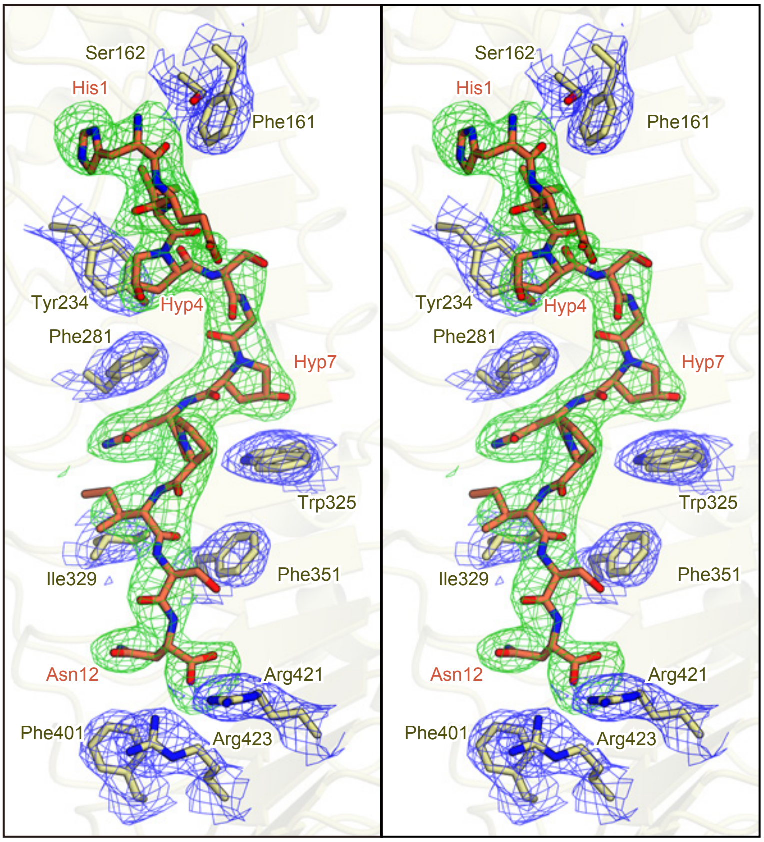

Supplementary Figure 3. Electron density maps of the TDIF-binding site.
Stereo views of the electron density maps of the TDIF-binding site of TDR. The *F*o – *F*c omit map calculated without TDIF (contoured at 3.0 σ) is shown as a green mesh, while the 2*F*o – *F*c electron density map (contoured at 1.5 σ) is shown as a blue mesh. TDIF and TDIF-interacting TDR residues are shown as orange and green sticks, respectively.

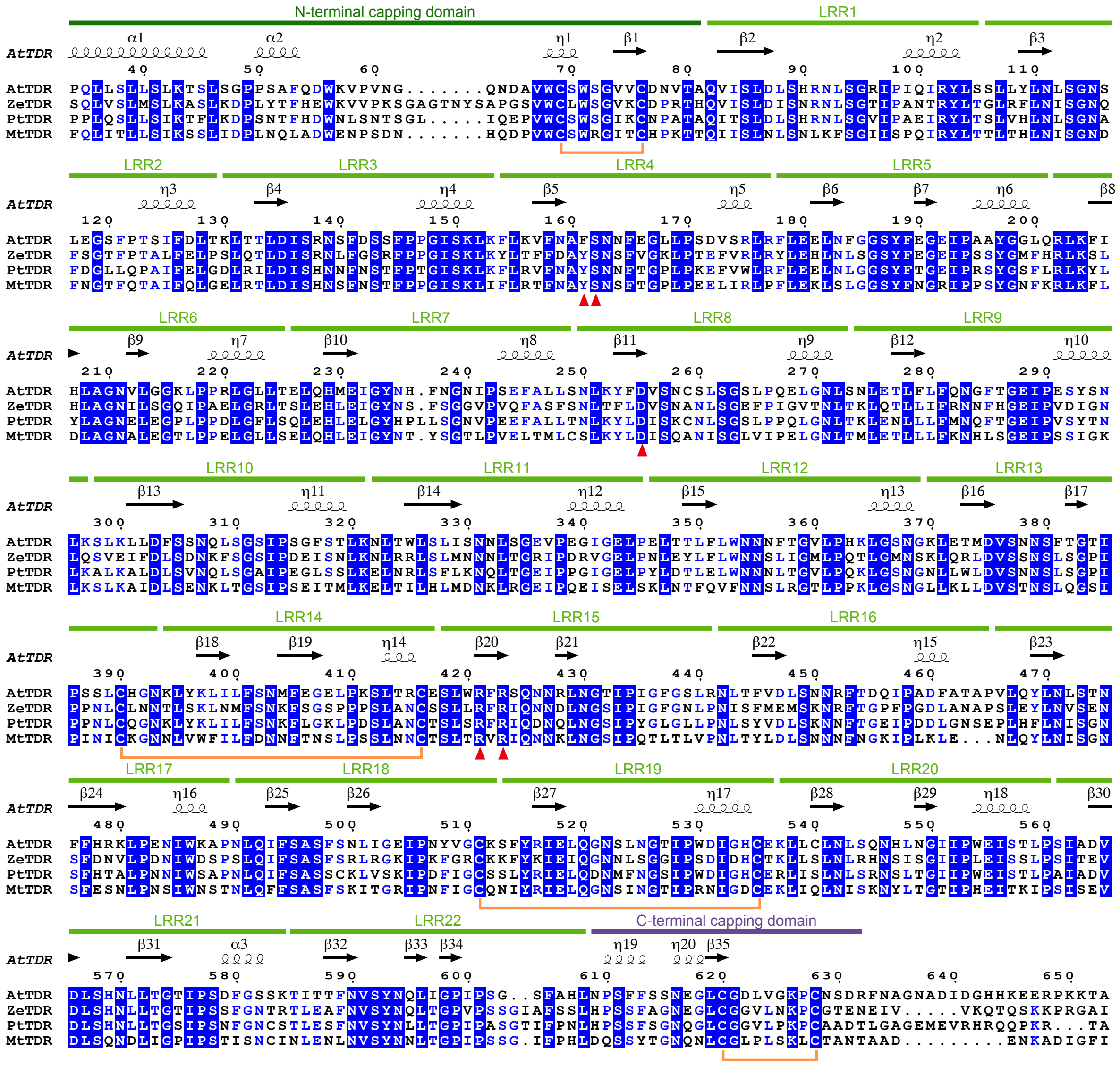

Supplementary Figure 4. Multiple sequence alignment of the extracellular domains of TDRs from different plant species. "At", "Ze", "Pt" and "Mt" represent *Arabidopsis thaliana*, *Zinnia elegans*, *Populus trichocarpa* and *Medicago truncatula*, respectively. Residues involved in TDIF recognition are indicated by red triangles. Cys residues that form disulfide bonds are indicated by orange lines.

a

b

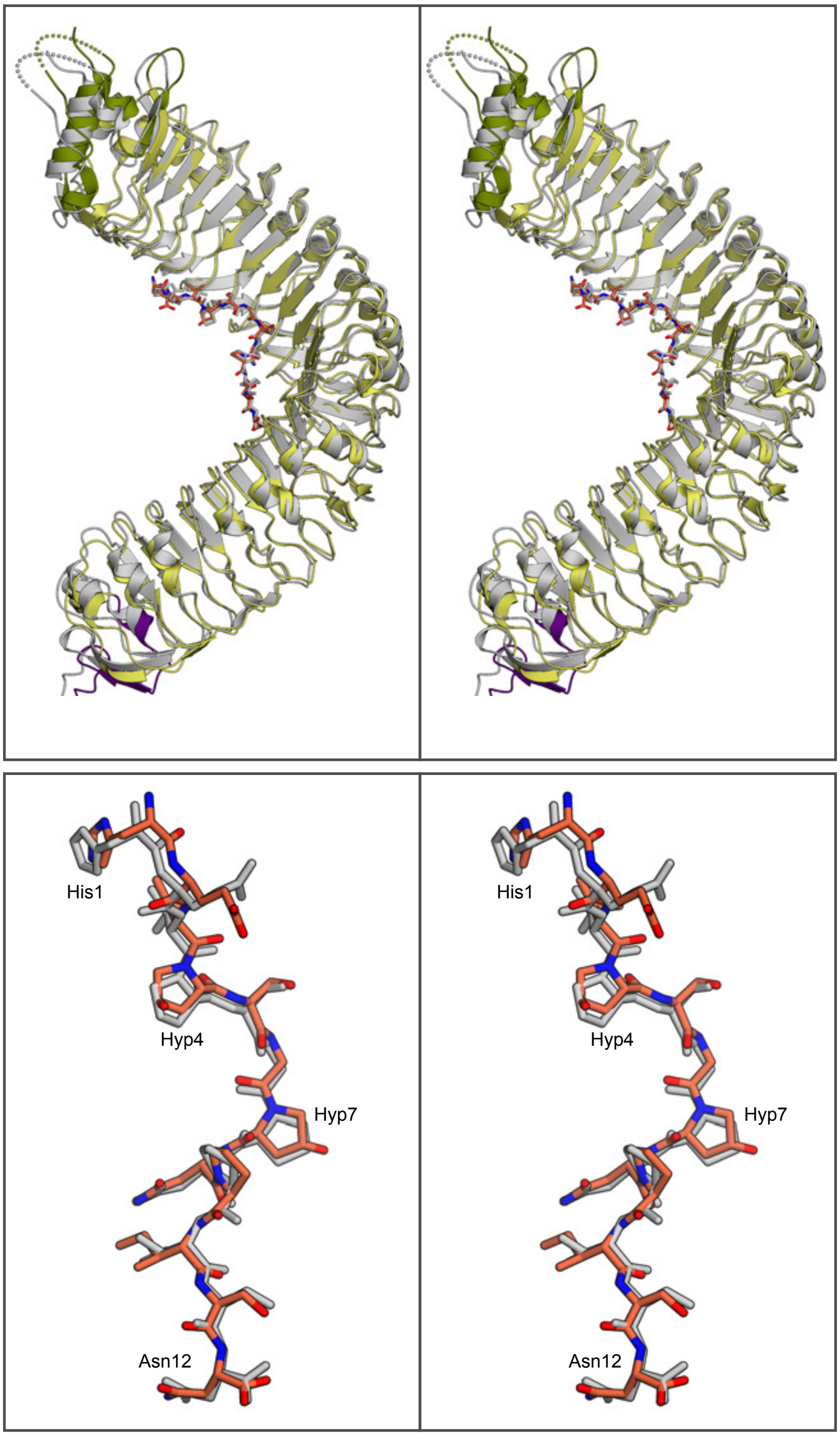

Supplementary Figure 5. Stereo views of the superimpositions of our TDR structure (colored) and the reported TDR structure (gray).
(a) Superimpositions of the TDR–TDIF complex structures. (b) Superimpositions of the TDIF peptide structures.

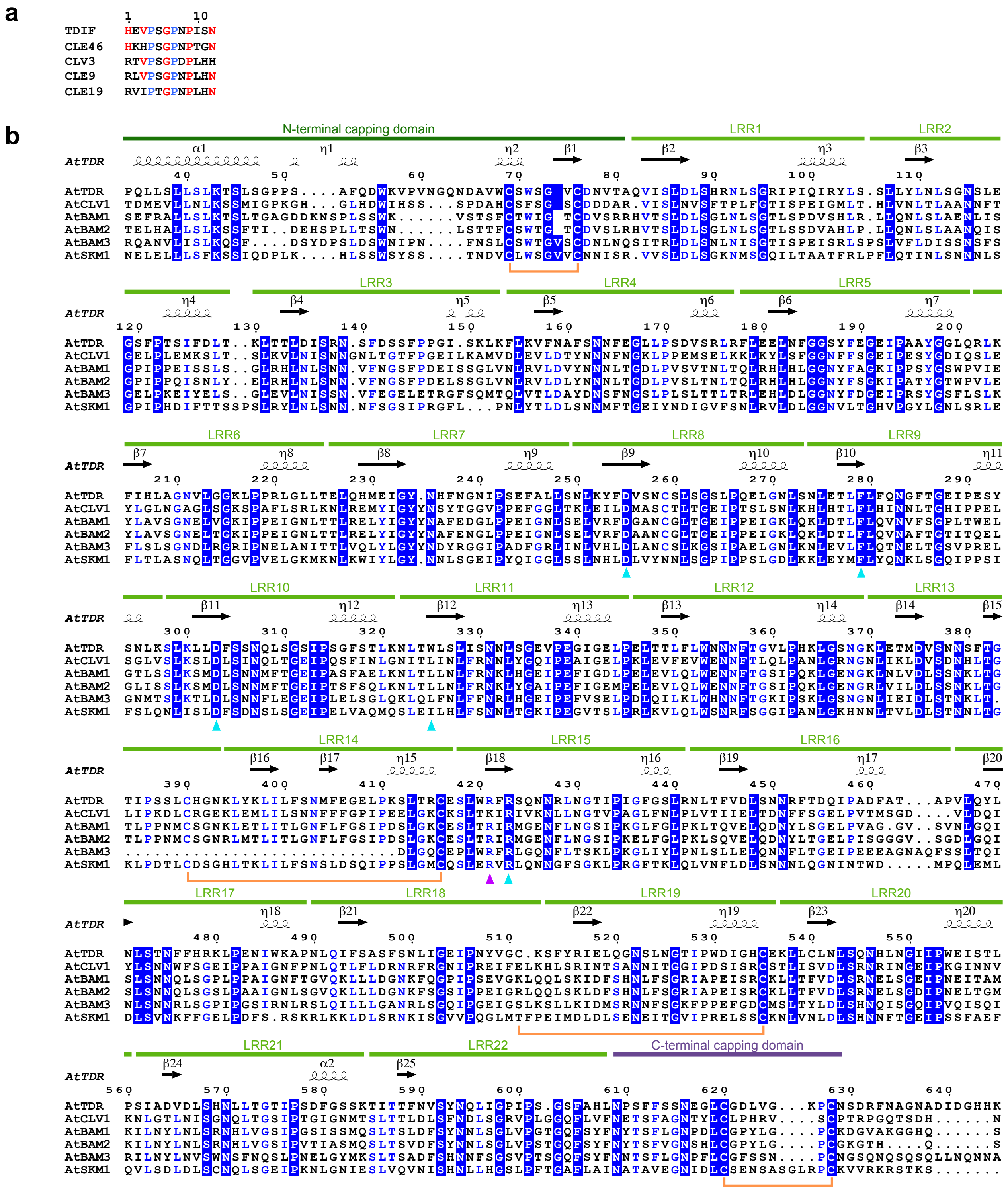

Supplementary Figure 6. Multiple sequence alignment of CLE family peptides and receptors in *Arabidopsis thaliana*.
(a) Sequence alignment of CLE family peptides. TDIF residues recognized by TDR are colored red. Conserved Pro/Hyp residues are colored blue. (b)Sequence alignment of the extracellular domains of LRR-RKs that perceive CLE peptides. Arg421 and residues involved in peptide backbone recognition are indicated by purple and cyan triangles, respectively.

Fig. 2e input

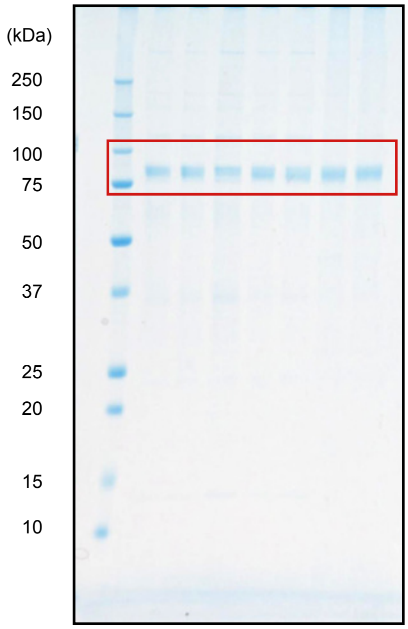

Fig. 2e pulldown

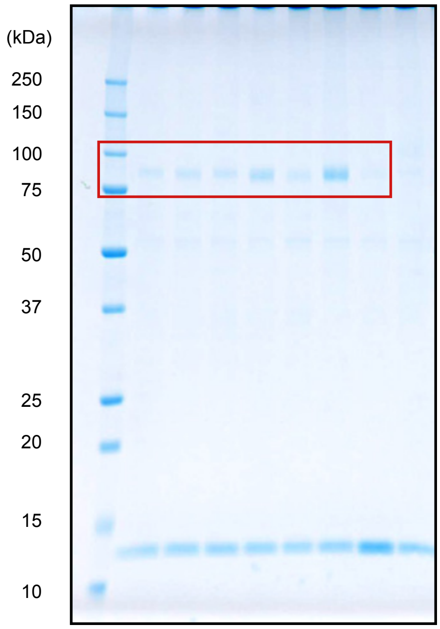

Supplementary Fig. 1

input pulldown

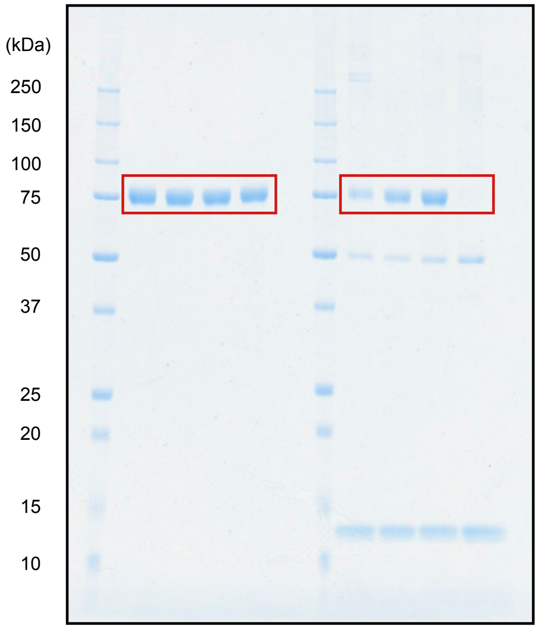

Supplementary Figure 7. Full images of SDS-PAGE gels.
Rectangles delimit cropped areas used in the indicated panels in Figure 2 and Supplementary Figure 1.
